# Optimized trans-cranial direct current stimulation for prolonged consciousness disorder in a patient with titanium mesh cranioplasty

**DOI:** 10.1007/s10072-024-07516-6

**Published:** 2024-04-09

**Authors:** Sun Im, Geun-Young Park, Tae-Woo Kim, Seong Hoon Lim

**Affiliations:** 1 Department of Rehabilitation Medicine, College of Medicine, Bucheon St. Mary’s Hospital, The Catholic University of Korea, Seoul, Republic of Korea; 2 Department of Rehabilitation Medicine, National Traffic Injury Rehabilitation Hospital, Gyeonggi-Do, Republic of Korea; 3 Department of Rehabilitation Medicine, Seoul St. Mary’s Hospital, College of Medicine, The Catholic University of Korea, Seoul, Republic of Korea; 4 Institute for Basic Medical Science, Catholic Medical Center, The Catholic University of Korea, Seoul, Republic of Korea

**Keywords:** Transcranial direct current stimulation, Minimal consciousness state, Non-invasive neuromodulation, Simulation, Optimization, MRI-based simulation, Consciousness

## Abstract

**Background:**

Transcranial direct current stimulation (tDCS) has been used for the restoration of awareness in patients with a minimal consciousness state (MCS). Most brains of patients in MCS may structurally and electrophysiologically differ from un-damaged brains. Moreover, tDCS is currently contraindicated for patients with craniotomy or skull with metallic implants.

**Case Presentation:**

We present a case with prolonged MCS over 1 year, who had severe brain damage, ventriculoperitoneal shunt, and cranioplasty with a titanium mesh, which was treated with tDCS which optimized with the simulation of the electric field based on the patient’s brain MRI. The patient was resulting in emergence from MCS. Six months later, she ate meals orally and started walking with assistance.

**Discussion and perspective:**

This personalized simulation based on MRI would make the treatment available even to patients with severe brain structural changes and metallic instrumentation.

## Introduction

Various treatment attempts have been explored for the treatment of prolonged state of minimal consciousness (MCS), including pharmacological agents such as dopaminergic agents, zolpidem, or applying neuromodulation. Transcranial direct current stimulation (tDCS) has shown promising results in MCS [1]. After that, a recent randomized trial did not reach an overall significant response but revealed some positive results in a subgroup in MCS and with traumatic etiology. Overcoming the current state of research needs another step in technical improvement [2].

The brains of most patients in a minimal conscious state (MCS) undergo severe damage, resulting in significant structural and electrophysiological differences when compared to an undamaged brain. Thus, neuromodulation with the conventional EEG 10–20 system has several neurophysiological limitations for these patients. Conventional tDCS is currently contraindicated for patients with craniotomy or skull with metallic implants. We present our novel first tDCS trial for a patient with prolonged MCS and severe brain damage, who had undergone several brain surgical procedures with titanium mesh cranioplasty but was treated with tDCS with simulation after a novel optimization approach based on using the patient’s brain magnetic resonance image (MRI). The treatment demonstrated remarkable safety and success, leading to the patient’s emergence from MCS accompanied by substantial neurological improvement.

## Case

**Fig. 1:**
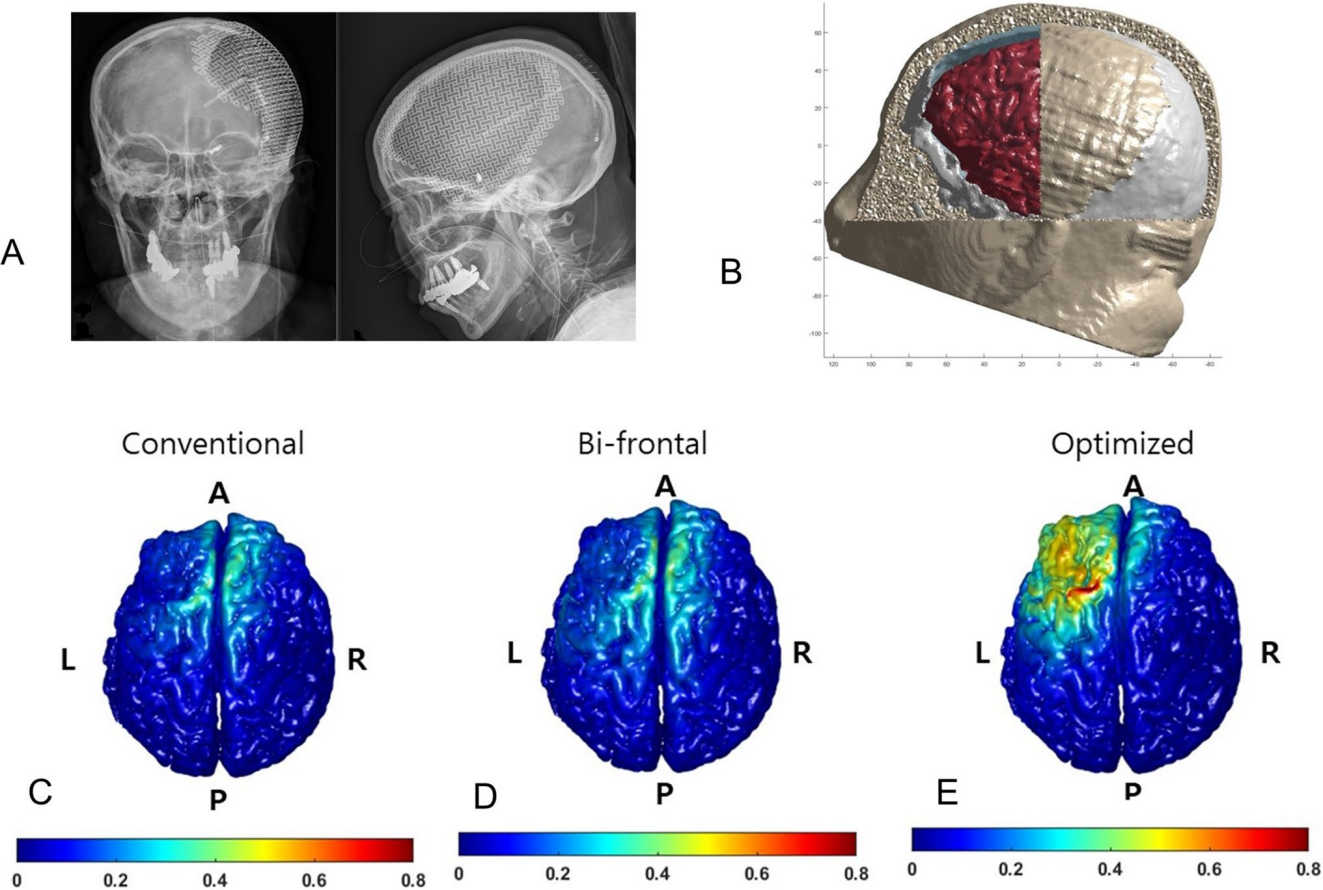
Simulation and the locations by conventional and optimization for targetting the right dorsolateral prefrontal cortex. The red pad is the cathode, and the blue pad is the anode. **A** Anteroposterior and lateral plain X-rays. The X-rays demonstrate the titanium mesh covering the left cranium. **B** The simulation montages used to simulate the effects on cortical current flow from tDCS. **C**–**E** Simulation shows that the Enorm field values of the right dorsolateral prefrontal cortex were stimulated by tDCS via three methods. **C** Simulation via conventional stimulation with 10–20 system. **D** Simulation via bi-frontal cortex stimulation. **E** Simulation via optimization based on the patient’s MRI. A, anterior; P, posterior; R, right; and L, left

A 62-year-old woman underwent a series of operations for subarachnoid hemorrhage with a ruptured posterior communicating artery, including coil embolization, decompressive craniectomy, ventriculoperitoneal shunt, and cranioplasty with a titanium mesh (Fig. 1A). Following the operations, the patient was diagnosed with MCS, and her revised coma recovery scale (CRS-r) score was 3 (A0V0M2O0C0Ar1) [3]. One year later due to failure of emergence of MCS, attempts for non-invasive neuromodulation treatment to improve the patient’s level of consciousness were discussed. However, due to the skull defect and titanium implant, conventional neuromodulation with tDCS was not considered a treatment option [4].

Under the consensus and agreement from all her legal guardians, she underwent treatment with tDCS to facilitate the dorsolateral prefrontal cortex (DLPFC). We simulated the electric field and locations of stimulation for targeting the right DLFCS via Neurophet®’s tESLab based on the patient’s MRI [5]. The simulation montages and electric field for stimulations with optimization based on 1-mm MRI resolution showed the proper Enorm of the right DLPFC (Fig. 1B–E). Treatment with tDCS was performed according to the following protocol; the cathode and anode were positioned as shown in Fig. 1F. A current intensity of 2 mA and a simulation time of 30 min were applied. Treatment with tDCS was performed according to the following protocol; the cathode and anode were positioned as shown in Fig. 1F. A current intensity of 2 mA and a simulation time of 30 min were applied. The tDCS intervention was performed 10 times for 2 weeks. The patient received in conjunction conventional rehabilitation therapy, such as physical and occupational therapy for 2–4 h per day (5 days per week). The therapies were performed passively, i.e., not in a goal-oriented manner, due to the debilitating nature of DOC. After the patient was treated with ten consecutive optimized tDCS sessions, the follow-up CRS-r score showed improvement to 17 (A2V4M3O3C2Ar1). Two months later, she showed dramatic neurological recovery, underwent successful nasogastric tube weaning, and was able to start full oral feeding. Six months later, she started walking with assistance.

## Discussion and perspective

The conventional 10–20 system has served as a widely adopted method for non-invasive neuromodulation. Nevertheless, it possesses a limitation in that it only offers an approximate estimation of the targeted location. Moreover, targeting becomes more challenging in a damaged brain after a stroke or traumatic brain injury. After sustaining injury, the brain initially swells, leading to an increase in size and a decrease in electrical resistance. Over time, the brain shrinks due to leukomalacia, which increases electrical resistance as a result of astrocytosis. In recent years, medical treatments have shifted from generalized and blinded to personalized and guided approaches. Studies have shown that blinded treatment with the 10–20 system for primary motor cortex using repetitive transcranial magnetic stimulation (rTMS) failed to produce statistical therapeutic effects on motor function in patients with subacute stroke, while guided rTMS using 120-channel EEG for refractory epilepsy resulted in a reduction in epilepsy frequency [6, 7]. The skull and scalp act as high-frequency and low-frequency filters [8]. Most neurosurgical procedures leave a scar, which can alter these filters. In this case, subarachnoid hemorrhage and several neurosurgical procedures altered the brain structure, while the presence of shunt and titanium mesh cranioplasty may give further disruption of the filters. After some brain surgery, the conductivity of large skull defects/plates might be increased; current is shunted away from the directly underlying cortex and concentrated in the cortex underlying the defect perimeter [9]. Thus, neuromodulation with tDCS in these cases can lead to uneven current flow with unpredictable and potentially undesirable stimulation effects. Our simulation results provided the calculated Enorm field and appropriate positioning for stimulation to overcome these setbacks (Fig. 1C–E). This case report provides evidence that patients with metallic surgical procedures can undergo successful tDCS treatment with dramatic neurological recovery after proper optimization based on a personalized and guided approach.

In summary, recent advances in segmentation and stimulation procedures have made tDCS treatment possible even in complex cases involving patients with metallic implants. The growing popularity of non-invasive neuromodulation highlights the importance of novel optimization and personalization approaches to ensure the effective use of noninvasive brain stimulation treatments in these patients. Our case offers compelling evidence that this innovative approach holds good potential in establishing tDCS as a safe treatment option with predictable clinical results, regardless of the extent of structural changes or the presence of metallic implants.

## Data Availability

The data presented in this study are available on request from the corresponding author.
