# Correction to: Optimized trans‑cranial direct current stimulation for prolonged consciousness disorder in a patient with titanium mesh cranioplasty

**DOI:** 10.1007/s10072-024-07636-z

**Published:** 2024-06-08

**Authors:** Sun Im, Geun‑Young Park, Tae‑Woo Kim, Seong Hoon Lim

**Affiliations:** 1 Department of Rehabilitation Medicine, College of Medicine, Bucheon St. Mary’s Hospital, The Catholic University of Korea, Seoul, Republic of Korea; 2 Department of Rehabilitation Medicine, National Traffic Injury Rehabilitation Hospital, Gyeonggi‑Do, Republic of Korea; 3 Department of Rehabilitation Medicine, College of Medicine, Seoul St. Mary’s Hospital, The Catholic University of Korea, Seoul, Republic of Korea; 4 Institute for Basic Medical Science, Catholic Medical Center, The Catholic University of Korea, Seoul, Republic of Korea

**Correction to: Neurological Sciences (2024)**

10.1007/s10072-024-07516-6

The original article requires updates to the Funding section. The updated Funding is shown below.

**Funding** This work was supported by the National Research Foundation of Korea funded by the Korean government (No. NRF 2021R1A2C1012113), the Promotion of Innovative Business for Regulation-Free Special Zones funded by the Ministry of SMEs and Startups (MSS, Korea) (P0020624).

The original article has been corrected.

